# Folate-targeted immunotherapy effectively treats established adjuvant and collagen-induced arthritis

**DOI:** 10.1186/ar1944

**Published:** 2006-04-28

**Authors:** Chrystal M Paulos, Bindu Varghese, William R Widmer, Gert J Breur, Erina Vlashi, Philip S Low

**Affiliations:** 1 Department of Chemistry, 560 Oval Drive, 1393 Brown Bldg., Purdue University, West Lafayette, IN 47907-1175, USA; 2 Department of Veterinary Clinical Sciences, School of Veterinary Medicine, Purdue University, West Lafayette, IN 47907-1175, USA

## Abstract

Activated macrophages express a cell surface receptor for the vitamin folic acid. Because this receptor is inaccessible or not measurably expressed on other normal cells, folic acid has been recently exploited to selectively deliver attached radio-emitters to sites of activated macrophage accumulation, allowing scintigraphic imaging of inflamed joints and organs of arthritic rats. We demonstrate here that folate-linked haptens can also be targeted to activated macrophages, decorating their cell surfaces with highly immunogenic molecules. Under conditions in which the rodent has already been immunized against keyhole limpet hemocyanine-(fluorescein isothiocyanate) FITC, activated macrophages are eliminated. Administration of folate-FITC conjugates to rodents with experimental arthritis attenuates (a) systemic and peri-articular inflammation, (b) bone and cartilage degradation, and (c) arthritis-related body weight loss. Treatment with folate-hapten conjugates is comparable to methotrexate, etanercept, anakinra, and celecoxib at alleviating the symptoms of arthritis. We conclude that reduction of activated macrophages by folate-targeted immunotherapy can ameliorate the symptoms of arthritis in two rodent models of the disease.

## Introduction

Rheumatoid arthritis (RA) is an autoimmune disease characterized by inflammation of the joints and destruction of cartilage and bone. Activated macrophages have been identified as a key mediator of the disease, as numbers and level of macrophage activation correlate with the extent of joint inflammation and bone degradation [1-4]. In addition, neutralization of inflammatory mediators secreted by activated macrophages suppresses symptoms of the disease [1-5].

Many therapies for RA are specifically designed to eliminate pro-inflammatory byproducts of activated macrophages [6]. Remicade and etanercept neutralize tumor necrosis factor alpha (TNF-α) [7-9], whereas anakinra blocks the activity of interleukin (IL)-1 [8-10]. Celecoxib and Vioxx are inhibitors of cyclooxygenase-2 [11], and anti-oxidants inactivate reactive oxygen species. Yet some patients require other remedies, either because of the ineffectiveness of the above therapies or due to their associated toxicities [12,13].

Efforts have been made to eliminate the entire population of macrophages [14-19], but elimination of all mononuclear phagocytes can be harmful, because they are important in fighting infectious diseases and promoting tissue repair [20,21]. Thus, an attractive alternative to elimination of all macrophages might be to remove only that subpopulation that promotes RA (that is, the activated macrophage).

Activated macrophages from patients and rodents with arthritis over-express a cell surface receptor for folic acid that also binds folate-linked compounds with high affinity (K_D _approximately 10^-10 ^M) [22-24]. Recent studies have shown that folate-linked radiopharmaceuticals concentrate in arthritic joints, enabling visualization of such tissues by gamma scintigraphy [23-25]. We hypothesized therefore that selective removal of activated macrophages with folate-linked drugs could be exploited to treat RA with little toxicity to other tissues. Here, we report a test of this strategy in two distinct rodent animal models of the disease.

## Materials and methods

Heat-killed *Mycoplasma butyricum *(BD Biosciences, Sparks, MD, USA); light mineral oil, methotrexate (MTX), clodronate, bovine serum albumin, keyhole limpet hemocyanine (KLH), anti-rat immunoglobulin G (IgG)-horseradish peroxidase (HRP), IgG-phycoerythrin conjugates, and alum (Sigma, St. Louis, MO, USA); biotin-conjugated goat anti-rat IgG1, IgG2a, and IgG (H+L), and streptavidin-conjugated HRP (Caltag Laboratories, Burlingame, CA, USA); aminofluorescein (single isomer) and fluorescein isothiocyanate (FITC) (Molecular Probes, Eugene, OR, USA); Microcon-30 membranes (Millipore Corp., Bedford, MA, USA); TiterMax Gold^® ^adjuvant (CytRx Corporation, Los Angeles, CA, USA); Celecoxib (Pfizer, Ann Arbor, MI, USA); entanercept and anakinra (Amgen, Inc., Thousand Oaks, CA, USA); EC20 (a folate-linked chelator of ^99m^Tc) and folate-FITC (Endocyte, Inc., West Lafayette, IN, USA) were obtained from commercial sources. The studies were approved by the Purdue University Animal Care and Use Committee.

### Induction and detection of anti-FITC antibodies

Anti-FITC antibodies were induced in rodent models with experimental arthritis by vaccination with KLH-FITC (KLH-FITC molar ratio of 1:13), using a modification of a published procedure [26]. Rodents were immunized subcutaneously with an emulsion of 150 μg KLH-FITC/200 μl adjuvant comprised of either TiterMax Gold^® ^(two injections each on days 0 and 28) or Alum (three injections each on days 0, 14, and 28). Ten days after the last boost, the serum was analyzed for anti-FITC antibodies by enzyme-linked immunosorbent assay [26].

### Induction and monitoring of experimental arthritis in rodents

Adjuvant-induced arthritis (AIA) was promoted in 200-g female Lewis rats (Charles River Laboratories, Wilmington, MA, USA) via either the footpad method [27] or the base-of-tail method [8]. Collagen-induced arthritis (CIA) was initiated in male DBA/1 *LacJ *mice (approximately 7 weeks old) (Chondrex, Inc., Redmond, WA, USA). The arthritic rodents were weighed weekly. Arthritis scores were determined using a weighted criterion (Chondrex, Inc.) and scored by a trained investigator blinded to the treatment groups. When the arthritis score reached 7, mice were randomly assigned to different treatment groups. Rodents were maintained on a folate-deficient diet (Harlan Tec) for 3 weeks prior to each study to lower serum folate levels to their physiologic range (approximately 25 nM) [28].

### Folate-targeted immunotherapy in experimental arthritis

Folate-FITC was administered i.p. to KLH-FITC-immunized rodents according to the doses/schedules described in each figure legend. For negative controls, KLH-FITC-immunized rodents were treated with the non-targeted aminofluorescein, which displays no affinity for folate receptors. Alternatively, non-immunized rodents were treated with folate-FITC, which binds readily to FR^+ ^cells but cannot mediate anti-FITC antibody binding in the absence of KLH-FITC immunization. Folate-targeted immunotherapy (FTI) was compared with other therapies by treating AIA rats (base-of-tail method) 7 days after arthritis induction with one of the following: FTI (KLH-FITC + Folate-FITC, 300 nmole/kg per day, i.p.), MTX (0.75 mg/kg per week, i.p.) [29], celecoxib (20 mg/kg, oral gavage every other day) [11], etanercept (4 mg/kg per day, i.p.) [7-9], clodronate liposomes (3.6 mg/kg on days 8, 16, and 23, i.p.) [16], or anakinra (1.5 mg/kg per hour, continuous infusion via subcutaneously implanted pump; Alzet, Cupertino, CA, USA) [8-10].

### Evaluation of therapeutic potencies

To determine whether FTI could ameliorate the symptoms of experimental arthritis in rodent models, disease status was assessed by monitoring changes in limb volume/ankle diameter, radiological score (RAD score), and systemic inflammation. Limb volume was determined by calculating the product of the measured length, width, and height of the limb (average ± SD, 8 rats/group). To determine the impact of the therapies on bone/cartilage degradation, lateral radiographic projections of the tarsus of each rat were scored at the end of each study. Radiographs were taken with direct exposure (1:1) on un-screen KODAK X-OMAT TL film (Kodak, Rochester NY, USA) using a Faxitron X-ray system with a 0.5-mm focal spot and beryllium window (Faxitron X-ray Corporation, Wheeling, IL, USA). Radiographs were scored by a board-certified veterinary radiologist blinded to the treatment groups. All radiographs were evaluated by a board-certified radiologist without knowledge of the assignment of treatment groups. RAD scores were assigned according to a modification of a previously described method [27]. The radiographic changes were graded numerically according to severity: increased soft tissue volume (0–4), narrowing or widening of joint spaces (0–5), subluxation (0–3), subchondral erosion (0–3), periosteal reaction (0–4), osteolysis (0–4), and degenerative joint changes (0–3).

### Analysis of FR^+ ^macrophages

To compare the abundance of macrophages in the ankle joints of arthritic rats that were treated with FTI versus phosphate-buffered saline (PBS), sections were analyzed by immunohistochemistry for ED1 antigen using a protocol described previously [30]. Briefly, un-injected hind paws (ankle joint extending from the distal portion of the tibia to the medial metatarsals) were fixed in 2% paraformaldehyde for 24 hours. They were then washed with PBS overnight at 4°C and decalcified in 10% EDTA for 3 weeks at 4°C. After decalcification, specimens were washed in PBS, and 10-μm thick sections were cut serially with a cryostat. Sections were prepared from the middle part of each ankle joint. A modified staining procedure was used [30]. Sections were incubated with anti-ED1 (1:100; Serotec, Inc., Raleigh, NC, USA) in a humidified chamber overnight (4°C) followed by peroxidase-conjugated secondary antibody (1:50, STAR72; Serotec, Inc.) for 1 hour at room temperature. To determine whether other therapies could also reduce the number of FR^+ ^activated macrophages, scintigraphy and the biodistribution of a folate-^99m^Tc radioimaging agent (EC20) in relevant tissues were also evaluated [23].

### Analysis of the long-term toxicity of FTI

To obtain an indication of the toxicity associated with treatment with FTI, KLH-FITC-immunized rats were injected three times per week for 15 weeks with PBS or folate-FITC (300 nmole/kg). Four rats per group were sacrificed every 3 weeks, and blood was analyzed for indicators of organ function (Analytics, Inc., Bethesda, MD, USA). Solid tissue samples were obtained from each animal, and organs were examined histologically for signs of tissue damage (Purdue Animal Disease and Diagnostic Laboratory).

### Splenomegaly

As documented elsewhere [27], one diagnostic characteristic of systemic inflammation in AIA is a gradual increase in spleen weight to more than twice its normal value. Therefore, to estimate the impact of the various therapies on systemic inflammation, the weight of each animal's spleen was measured at the end of each study.

## Results

### FTI reduces localized and systemic folate-binding macrophages

**Figure 1:**
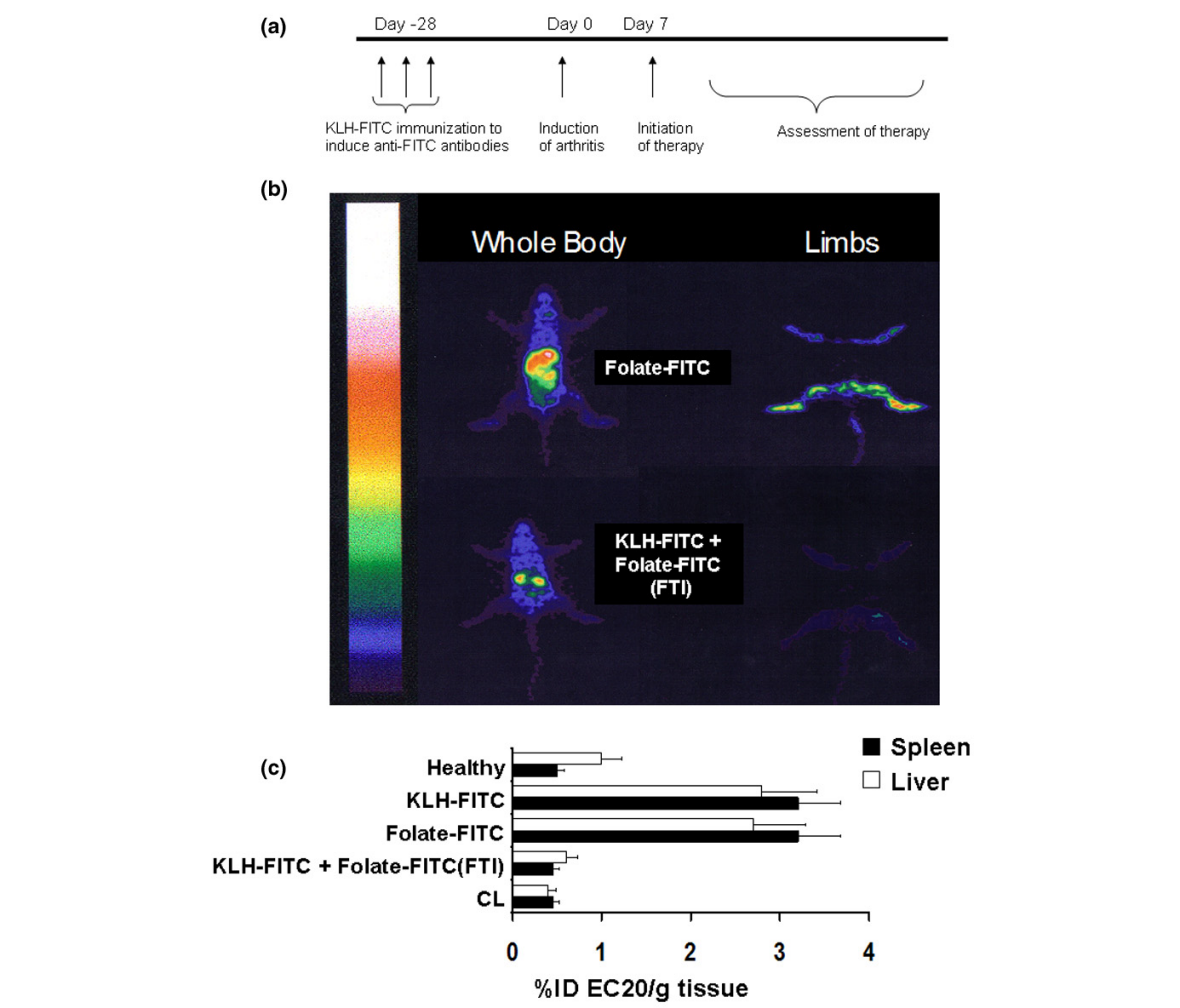
Folate-targeted immunotherapy reduces activated macrophages systemically in adjuvant-induced arthritic rats. **(a) **Treatment schedule for FTI. **(b) **The level of activated macrophages in internal organs (left) and limbs (right) of non-immunized arthritic rats (upper panel) and keyhole limpet hemocyanine-fluorescein isothiocyanate (KLH-FITC)-immunized arthritic rats (lower panel) 25 days after initiation of treatment with folate-FITC (375 nmole/kg twice a week until day 21) was imaged 4 hours after i.p. injection of 0.5 mg EC20. **(c) **Activated macrophage accumulation in the liver and spleen was evaluated by measuring EC20 uptake in healthy non-arthritic rats (Healthy), AIA rats immunized with KLH-FITC and left untreated (KLH-FITC), non-immunized rats injected i.p. with folate-FITC (375 nmole/kg twice a week), KLH-FITC-immunized AIA rats treated with folate-FITC (KLH-FITC + Folate-FITC [FTI], 375 nmole/kg twice a week), and AIA rats injected with clodronate liposomes ([CL]; 3.6 mg/kg on days 8, 16, and 21, i.p.). EC20 biodistribution was measured by removal of the indicated tissues, weighing and counting them for radioactivity; represented as percent injected dose of EC20 per gram tissue, for instance, %ID EC20/g tissue (mean ± standard deviation, *n *= 5 rats/group). Data are representative of three independent experiments. (c) folate-FITC versus KLH-FITC + folate-FITC (FTI), *p *< 0.001.

With the ability of folic acid to deliver attached imaging agents specifically to activated macrophages in rodents with experimental arthritis well established [23-25], the question naturally arose whether therapeutic drugs might also benefit from FR-specific targeting. Initial consideration of an appropriate drug for delivery as a folate conjugate led to re-examination of strategies already developed for the treatment of FR^+ ^tumors. Thus, it has been demonstrated that some cancer cells over-express a folate receptor and that the surfaces of cancer cells can be decorated with highly immunogenic haptens by targeting their FRs with folate-hapten conjugates [26]. Under conditions in which the rodent has already been immunized against the hapten, hapten-decorated cancer cells rapidly bind anti-hapten antibodies, resulting in their removal by FcR-expressing immune cells. Given that folate-targeted haptens can lead to eradication of FR^+ ^tumors, it seemed logical to explore whether a similar approach (Figure 1a) might be exploited to reduce FR-expressing activated macrophages.

AIA was generated in Lewis rats by footpad injection of an oil emulsion of heat-killed *M. butyricum*. Swelling of the injected limb began immediately, whereas swelling of the non-injected hind limb was more gradual, beginning approximately seven days after injection and continuing unabated until euthanasia became necessary. To determine whether FTI depletes folate-binding macrophages in AIA rats, we quantitated the uptake of a folate-linked chelate complex of ^99m^Tc (termed EC20) that was previously shown to be taken into activated macrophages by FR-mediated endocytosis [23]. Because accessible folate receptors are found in measurable numbers only on activated macrophages [22], cancer cells [26], and the proximal tubule cells of the kidneys [31], EC20 uptake in tumor-free liver, spleen, lungs, intestines, and other organs is roughly proportional to each organ's content of activated macrophages [23]. This uptake is visualized in both non-immunized and KLH-FITC-immunized arthritic rats after identical treatment with folate-FITC (Figure 1b). As evidenced from the γ-scintigraphic images, uptake of the folate-^99m^Tc complex (EC20) in spleen, liver, kidneys, and limbs is substantial in non-immunized rats treated with folate-FITC, but largely limited to the kidneys (which ubiquitously express the FR on the apical surface of their proximal tubules [31]) in KLH-FITC-immunized rats treated with folate-FITC (FTI).

To visualize inflammation in the limbs of arthritic rats, mid- and upper-body radiation was shielded with lead blankets and radioimages of hind limbs were acquired by gamma scintigraphy. As expected, uptake of the folate-targeted ^99m^Tc agent (EC20) in hind limbs was more intense in non-immunized than in KLH-FITC-immunized AIA rats treated with folate-FITC (FTI) (Figure 1b). Taken together, these results suggest that FTI depletes activated macrophages both locally and systemically in AIA rats.

To quantitatively assess the degree of systemic depletion of activated macrophages by FTI, uptake of EC20 was analyzed in the spleen and liver of AIA rats by scintillation counting. EC20 content was approximately seven times greater in the organs of both non-immunized rats treated with folate-FITC and KLH-FITC-immunized rats left untreated than in healthy rats or KLH-FITC-immunized animals treated with folate-FITC (FTI) (Figure 1c; *p *< 0.001). Similar suppression of EC20 uptake was also observed in arthritic rats treated with clodronate liposomes (CL), a bisphosphonate commonly used to deplete all mononuclear phagocytes [23,25,30]. This latter observation supports the contention that the EC20 uptake is mediated by activated macrophages, as previously shown [23,25]. Finally, FTI reduced AIA-induced spleen enlargement twofold relative to untreated rats, suggesting that the immunotherapy reduces systemic inflammation.

**Figure 2:**
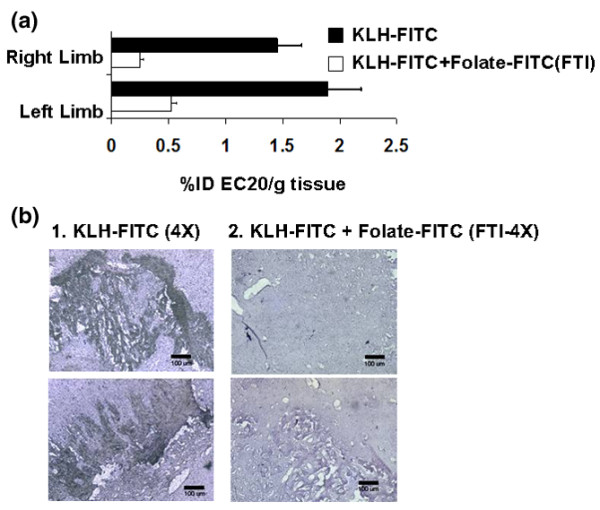
Folate-targeted immunotherapy reduces folate receptor expressing macrophages in inflamed tissue of AIA rats. **(a) **The level of folate receptor (FR)+ activated macrophages in the limbs of non-immunized (Folate-fluorescein isothiocyanate [FITC]) and keyhole limpet hemocyanine (KLH)-FITC-immunized AIA rats (KLH-FITC + Folate-FITC [FTI]) 25 days after initiation of treatment with folate-FITC was determined 4 hours after i.p. injection of 0.5 mg EC20. Folate-FITC was administered at 375 nmole/kg twice a week until day 23, and estimates of FR^+ ^macrophage numbers were made by weighing and counting the limbs for EC20 radioactivity (± standard deviation for *n *= 5 rats/group; [folate-FITC] vs. [KLH-FITC + folate-FITC (FTI)], *p *< 0.001). **(b) **Histological analyses were conducted to obtain a visual assessment of the abundance of ED1^+ ^macrophages remaining in the inflamed soft joint tissue of the ankle joints (inflamed soft tissue adjacent to bone) of AIA rats immunized with KLH-FITC and then treated with either phosphate-buffered saline (PBS) alone (KLH-FITC) or folate-FITC (KLH-FITC + Folate-FITC [FTI]). Representative images from the joint tissue of two PBS-treated and two folate-FITC (FTI)-treated rats are shown (*n *= 5 rats/group).

Consistent with the decrease in EC20 uptake in the limbs of AIA rats treated with FTI (KLH-FITC + folate-FITC) (Figures 1b and 2a; *p *< 0.001), a considerable reduction of ED1^+ ^(a macrophage-specific marker) cells was also demonstrated by immunohistochemical staining of the inflamed soft tissue of the ankle joint of the FTI-treated rats (see representative micrographs in Figure 2b). Taken together, the scintigraphic images, EC20 uptake analyses, and immunohistochemistry demonstrate that FTI reduces both systemic and articular accumulation of folate-binding macrophages in arthritic rats.

### FTI reduces bone and cartilage degradation and inflammation in limbs of AIA rats

**Figure 3:**
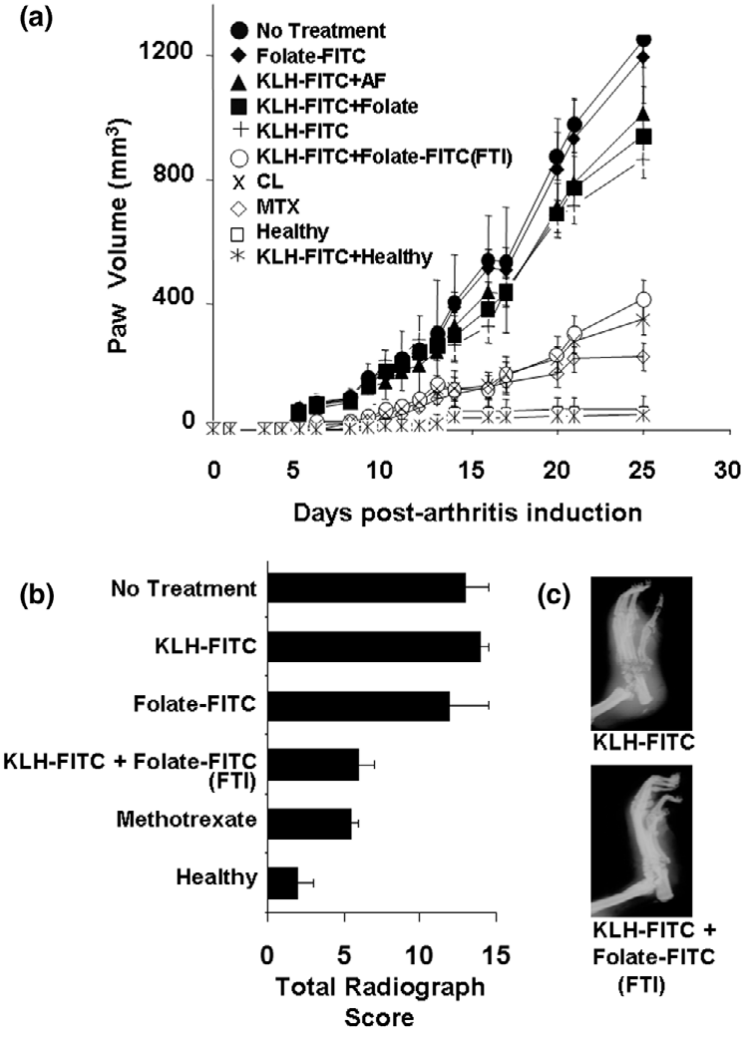
Effect of folate-targeted immunotherapy on joint inflammation and bone/cartilage destruction in AIA rats. Where indicated, AIA rats were immunized with a 1:1 suspension of keyhole limpet hemocyanine-fluorescein isothiocyanate (KLH-FITC) and TiterMax Gold adjuvant to generate a high anti-FITC antibody titer. On day 0, rats were induced to develop AIA via the footpad method. **(a) **Treatments began on the day of arthritis induction, and rats were given i.p. doses twice a week at 375 nmole folate-FITC/kg. Treatment groups included: AIA rats not immunized and not treated (No Treatment), non-immunized rats treated with folate-FITC (Folate-FITC), AIA rats immunized against fluorescein and treated with non-targeted aminofluorescein (KLH-FITC + AF), AIA rats immunized against fluorescein and treated with folic acid alone (KLH-FITC + Folate), AIA rats immunized against fluorescein but not further treated (KLH-FITC), AIA rats immunized against fluorescein and treated with folate-FITC (KLH-FITC + folate-FITC [FTI]), AIA rats treated on days 8, 16, and 23 with 3.6 mg/kg clodronate liposomes (CL), AIA rats treated with methotrexate (MTX; 0.75 mg/kg per week, i.p.), immunized healthy rats (KLH-FITC + Healthy), and untreated healthy rats (Healthy), respectively. Data are representative of two independent experiments (*n *= 5 rats/group; [folate-FITC] vs. [KLH-FITC + folate-FITC (FTI)], *p *< 0.01). The impact on bone and cartilage degradation was assessed both by analysis of radiological (RAD) scores **(b) **and visual inspection **(c) **on day 25 after arthritis induction (mean ± standard deviation, *n *= 4 or 5 rats/group). Data are representative of three independent experiments. (RAD scores: folate-FITC vs. KLH-FITC + folate-FITC (FTI), *p *< 0.05).

In the footpad arthritis model, AIA rats immunized against fluorescein (KLH-FITC) and then treated with folate-FITC (FTI) experienced a >60% reduction in swelling of non-injected limbs when compared with untreated arthritic rats (Figure 3a). Evidence that the observed resolution of symptoms depended on prior vaccination against fluorescein (KLH-FITC) was obtained from controls that showed no therapeutic benefit after treatment of non-immunized animals with folate-FITC (solid diamonds). The requirement for folate targeting was demonstrated by the fact that the therapy also failed when KLH-FITC-immunized rats were left either untreated or treated with non-targeted aminofluorescein (solid triangle). Finally, the observation that folic acid alone exhibited no significant effect on the KLH-FITC-immunized AIA rats (solid squares) argues that the vitamin's targeting function, rather than its role in metabolism, constitutes its major contribution to the immunotherapy. Importantly, this non-optimized FTI protocol reduced inflammation in arthritic limbs with similar efficacy to CL and MTX (Figure 3a).

By day 25 in AIA rats, histological analyses revealed multiple symptoms of established arthritis, including cartilage and bone erosion, and massive macrophage infiltration. In KLH-FITC-immunized AIA rats treated with folate-FITC (FTI), however, the degree of cartilage and bone damage was markedly reduced relative to that of KLH-FITC-immunized rats not treated with folate-FITC (RAD scores: folate-FITC vs. KLH-FITC + folate-FITC, *p *< 0.05) (Figure 3b,c). A similar protective effect was observed in arthritic rats treated with MTX. Control studies further confirmed that the FTI was both dependent on anti-FITC antibody and FR-mediated, because no significant reduction in bone score was seen in either non-immunized rats treated with folate-FITC or in KLH-FITC-immunized rats left untreated.

### FTI reduces inflammation and bone and cartilage degradation in limbs of CIA mice

**Figure 4:**
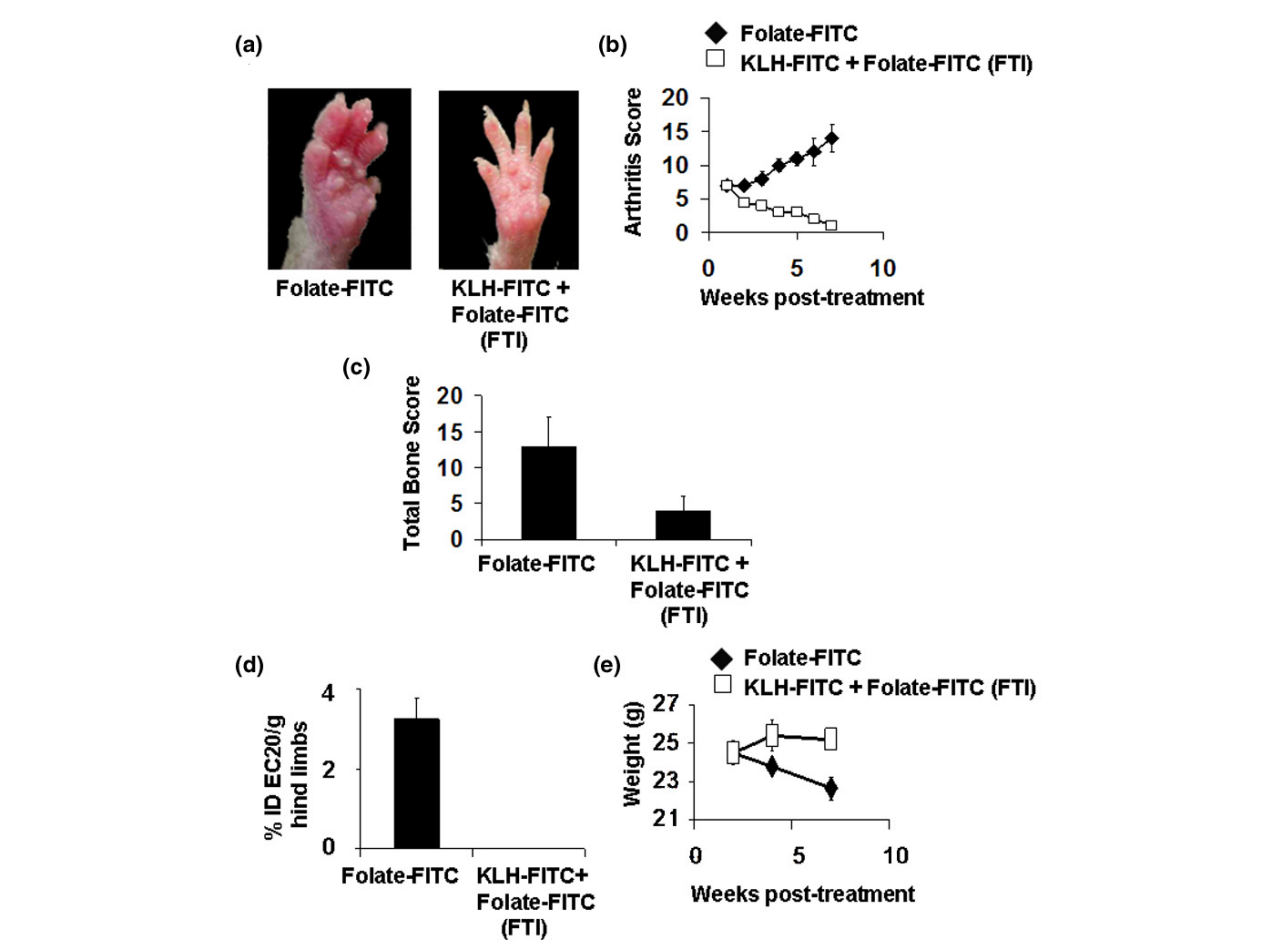
Effect of folate-targeted immunotherapy (FTI) on mice with established collagen-induced arthritis. Mice were immunized with keyhole limpet hemocyanine-fluorescein isothiocyanate (KLH-FITC) in an equal volume of TiterMax Gold adjuvant to generate a high anti-FITC antibody titer. Seven days after arthritis induction, when the average arthritis score had reached 7, mice were treated daily with 600 nmole/kg folate-FITC until day 38. **(a) **Differences between folate-FITC-treated and untreated mice were easily visualized. **(b) **Each limb was evaluated weekly by an individual blinded to the treatment groups and was assigned an arthritis score (see Methods and methods). **(c) **Bone erosion was analyzed on day 49 from radiographs by a radiologist blinded to the treatment groups. **(d) **Uptake of EC20 in both hind limbs was determined as a measure of activated macrophage accumulation on day 49, represented as percent injected dose of EC20 per gram hind limbs, for instance, %ID EC20/g hind limbs. **(e) **The change in weight of mice after arthritis induction was measured regularly.

Because therapeutic data in rodent models of arthritis can be model-specific, the efficacy of FTI was also evaluated in established CIA. All therapies were initiated seven days after onset of CIA, when disease symptoms had already progressed to a severe state. Visual inspection and arthritis scores (Figure 4a,b) revealed a marked reduction in inflammation after FTI. Furthermore, bone damage was lower in KLH-FITC-immunized mice treated with folate-FITC (FTI) than in similarly immunized mice not treated with folate-FITC (Figure 4c), and uptake of EC20 was also significantly lower in folate-FITC treated mice than in non-treated mice (Figure 4d). Importantly, CIA mice treated with FTI did not lose weight whereas vaccinated mice not treated with folate-FITC did (Figure 4e).

### FTI is comparable to other common RA therapies

For comparison with the potencies of other common arthritis therapies, a more stringent base-of-tail adjuvant-induced arthritis model was used [8]. The disease was allowed to develop 7 days prior to initiation of any therapy. Progression of AIA was evaluated continuously by measuring ankle diameter. Final disease status was assessed on day 42 by examining radiographs for articular bone and cartilage degradation and by determining EC20 uptake in macrophage-rich tissues. All therapies were dosed according to the optimal schedule and concentration reported by each manufacturer for use in rodents (see Materials and methods).

**Figure 5:**
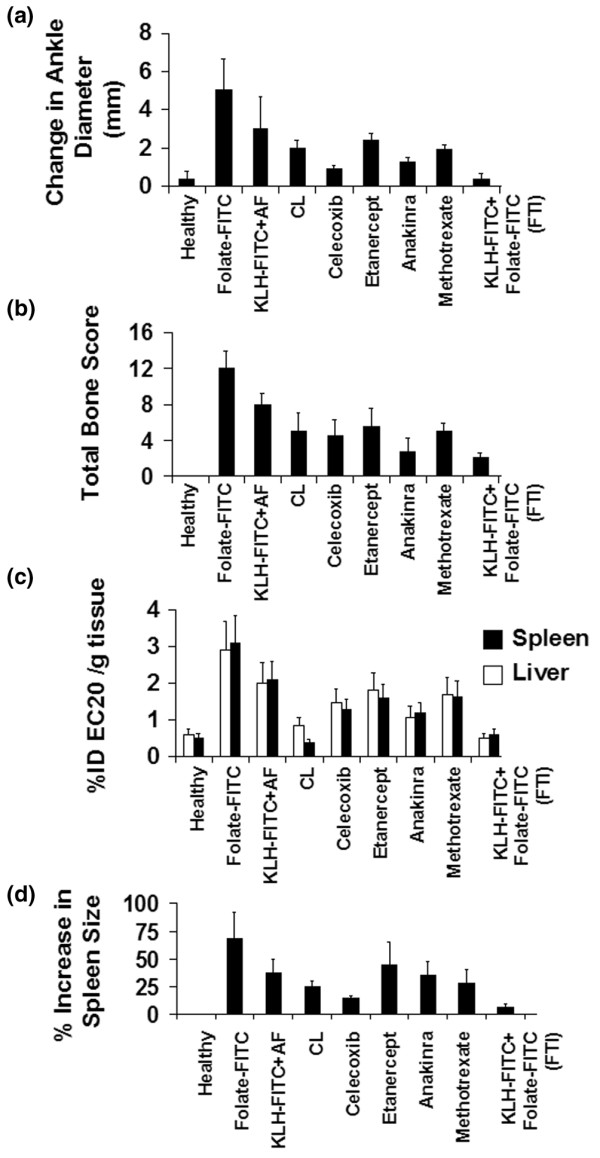
Comparison of the effects of various arthritis therapies on disease severity in rats with established adjuvant-induced arthritic (AIA). Rats were left either unimmunized or immunized with keyhole limpet hemocyanine-fluorescein isothiocyanate (KLH-FITC) in an equal volume of alum adjuvant to generate a high anti-FITC antibody titer. AIA was induced by the base-of-tail method, and 7 days after induction each therapy was initiated. The therapies were folate-FITC in non-immunized rats (folate-FITC; 30 nmole/kg per day, i.p.), non-targeted aminofluorescein (AF) in immunized rats (KLH-FITC + AF; 30 nmole/kg per day, i.p.), clodronate liposomes (CL; 3.6 mg/kg on days 8, 16, and 23, i.p.), celecoxib (20 mg/kg every other day, oral gavage), etanercept (4 mg/kg per day, i.p), anakinra (120 mg/kg per day, continuous infusion, Alzet osmotic pump), methotrexate (MTX; 0.75 mg/kg per week, i.p.), or folate-targeted immunotherapy (FTI) in immunized rats (KLH-FITC + folate-FITC [FTI]; 30 nmole/kg per day, i.p.). All groups were measured for arthritis symptoms by monitoring changes in ankle diameter **(a)**, bone and cartilage degradation **(b)**, uptake of EC20 in the spleen and liver **(c)**, and splenomegaly **(d)**. Data are representative of two independent experiments (mean ± standard deviation, *n *= 5 rats/group).

AIA rats treated with FTI showed a reduction in ankle diameter similar to that promoted by MTX, celecoxib, etanercept, anakinra, and CL (Figure 5a). Comparison of total bone scores (Figure 5b) also suggested that FTI was competitive with other therapies. Measurements of systemic inflammation, as indicated by uptake of EC20 in the liver and spleen (Figure 5c) and spleen enlargement (Figure 5d), further suggested that FTI systemically reduces the numbers of activated macrophages at least as effectively as most commercial treatments. In no case was the non-targeted aminofluorescein (KLH-FITC + AF) different from controls, reinforcing the need to target the hapten to achieve a response.

### FTI shows no signs of toxicity during 15 weeks of continuous treatment

**Figure 6:**
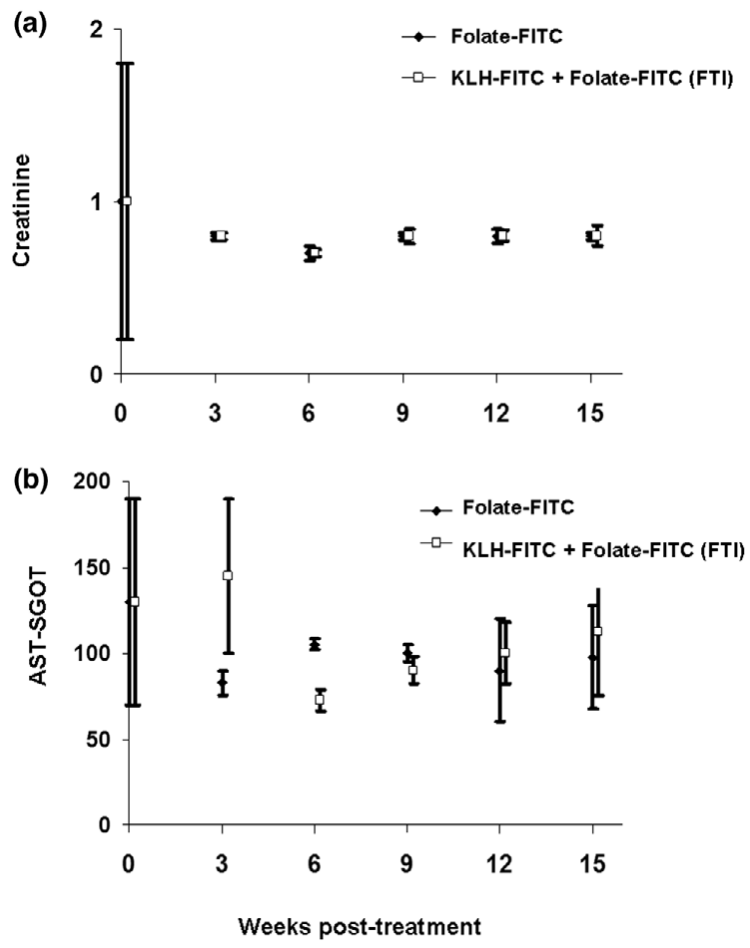
No indication of toxicity was detected in rats treated with folate-targeted immunotherapy for 15 weeks. Toxicity analysis associated with continuous treatment of healthy keyhole limpet hemocyanine-fluorescein isothiocyanate (KLH-FITC)-immunized rats with folate-targeted immunotherapy (FTI) for 15 weeks. Non-immunized or KLH-FITC-immunized rats were treated with folate-FITC (300 nmole/kg, three times per week) and sacrificed at the indicated times. Serum was analyzed for indicators of kidney (creatinine) **(a) **and liver (aspartate aminotransferase [AST-SGOT])**(b) **function. Tissue samples, including liver, kidney, brain, lungs, and bone marrow, were simultaneously submitted for histopathological analyses. No indication of toxicity was detected by either type of analysis (tissue section data not shown) (mean ± standard deviation, *n *= 8 rats/group).

To assess the long-term toxicity of FTI, both serum and tissues from non-immunized and KLH-FITC-immunized healthy rats treated three times per week for up to 15 weeks with folate-FITC were evaluated by a commercial pathologist (Analytics, Bethesda, MD, USA). Indicators of kidney (creatinine) and liver (aspartate aminotransferase) function were within normal ranges for the duration of the study (Figure 6). Also, no evidence of either macroscopic or microscopic damage was observed in any tissue-thin sections, including multiple sections of the liver, kidneys, brain, and other major tissues.

## Discussion

We have explored a novel approach for the treatment of experimental arthritis (the redirection of the immune system to reduce endogenous activated macrophage, which is known to be involved in maintenance of RA) [1-5,32-34]. Thus, ever since it was observed that activated, but not resting, macrophages express a folate receptor [22], folate-linked drugs have been found to specifically target arthritic joints and sites of inflammation [23-25]. In this study, we have exploited folate's targeting ability to selectively decorate activated macrophages with immunogenic haptens that can mediate their removal in immunized hosts. Prior vaccination against the hapten was found to be necessary, because non-immunized animals did not benefit from treatment with folate-FITC. Folate targeting of the hapten was shown to be critical, because identical treatment with non-targeted aminofluorescein was totally ineffective. Fluorescein derivatization was also found to be essential, because administration of folic acid without an attached fluorescein was equally impotent. In contrast, vaccination with KLH-FITC followed by treatment with folate-FITC led to significant reduction in both local and systemic inflammation and to measurable diminution in bone and cartilage degradation in two advanced-stage models of RA.

We hypothesize that the efficacy of the FTI is related to its ability to promote systemic depletion of FR^+ ^macrophages. As noted above, activated macrophages constitute a likely orchestrator of inflammation and joint destruction in experimental arthritis [1-5,32-34]. Not only do activated macrophages release reactive oxygen species, collagenases, cathepsins, and other mediators of joint destruction, but they also recruit other immune cells by releasing pro-inflammatory cytokines (TNF-α, IL-1, and IL-6), leukotrienes, and prostaglandins [25]. Several investigators [22,23,25] have demonstrated that activated macrophages express significant numbers of cell-surface FR. Because folate-linked drugs bind avidly to these receptors and localize to inflamed joints of animals and humans with arthritis [23-25], it seems reasonable to posit that their uptake at sites of inflammation might stem from their high affinity for FR. Others have, in fact, established that synovial macrophages from patients with RA aggressively bind folate-FITC [22], and we have similarly demonstrated that folate-FITC uptake in inflamed joints of rodents with experimental arthritis occurs primarily in cells expressing both FR and macrophage markers [23]. Because treatment of both CIA mice and AIA rats leads directly to a significant reduction in folate-binding macrophages, we speculate that painting of activated macrophage cell surfaces with folate-FITC directly causes their elimination in anti-FITC-immunized rodents by antibody-dependent pathways. Studies are currently under way to test this mechanism.

Comparative experiments revealed that FTI was as effective as CL, MTX, etanercept, anakinra, and celecoxib at alleviating the symptoms of experimental arthritis. Furthermore, FTI appeared to be better than the above therapies at suppressing systemic inflammation, as measured by both accumulation of EC20 in liver and spleen and by reduction of inflammation-mediated spleen enlargement. This capability to reduce systemic inflammation may be important, because patients with RA have a reduced life expectancy that is caused by systemic, rather than articular, inflammation [35].

An attractive feature of the FTI approach lies in its apparent lack of toxicity. Because FRs are also expressed on the apical surfaces of a few normal epithelia (for example, the choroid plexus of the brain, bronchioalveolar cells of the lungs, and proximal tubules of the kidney), it might have been anticipated that immune responses would have been elicited against these tissues also. However, fluorescence micrographs show that sites of FR expression on normal epithelia are inaccessible to circulating antibodies [31], because the receptor invariably resides on the apical surface of the epithelium where it faces a lumen that is usually inaccessible to parenterally administered drugs. Thus, pathological analyses have not revealed any cytotoxicity or immune damage to normal tissues.

One concern that must accompany systemic depletion of activated macrophages relates to the possible decline in defense against pathogens. Although no infections/diseases were observed in rats maintained on the therapy for 15 continuous weeks, nor in RA dogs treated for more than 2.5 years (Paulos CP, Varghese B, Vlashi E, Low PS, Widmer WR, and Breur GJ), unpublished observations), sufficient time has not yet elapsed to conclude that long-term defects will not emerge during continuous treatment. Although resting macrophages and other immune cells might compensate for elimination of most activated macrophages, it will be important to examine chronically treated animals for deficiencies in tasks normally performed by activated macrophages. Where temporary deficiencies do arise, it might be sufficient to remove the patient from the therapy until the deficiency subsides.

A second potential concern might arise from the possible formation of multimeric immune complexes in circulation after injection of the folate-hapten conjugate. However, because folate-FITC contains only one antibody-binding site, it cannot mediate formation of multimeric immune complexes, even though monomeric IgG-folate-FITC complexes are readily observed in circulation. Thus, deposition of macroscopic immune complexes in non-targeted tissues has not constituted a source of observed toxicity.

## Conclusion

In summary, we have demonstrated that removal of folate-binding macrophages suppresses established arthritis in two animal models of the disease. Because little measurable toxicity accompanied the therapy, these data suggest that removal or inactivation of folate receptor-expressing macrophages might constitute an alternate approach for treatment of the pathology. Thus, in addition to the existing pantheon of drugs aimed at neutralizing secretion products of activated macrophages (that is, Remicade, Enbrel, Humira, Kineret, Vioxx, Celebrex, and so on), examination of folate-targeted drugs that can block the inflammatory activity of the same cell type would seem to warrant further scrutiny [25].

## Abbreviations

AIA = adjuvant-induced arthritis; CIA = collagen-induced arthritis; CL = clodronate liposomes; FITC = fluorescein isothiocyanate; FR = folate receptor; FTI = folate-targeted immunotherapy; HRP = horseradish peroxidase; Ig = immunoglobulin; IL = interleukin; KLH = keyhole limpet hemocyanine; MTX = methotrexate; PBS = phosphate-buffered saline; RA = rheumatoid arthritis; RAD score = radiographic score; TNF-α = tumor necrosis factor alpha.

## Competing interests

PSL has shares in Endocyte, Inc.

## Authors' contributions

CMP designed the project, carried out the animal studies, and drafted the manuscript. BV designed the project, carried out the biodistribution studies, and drafted the manuscript. WRW blindly scored radiographs and contributed intellectually to the project. GJB carried out scintigraphic imaging of the rodents and contributed intellectually to the project. EV carried out the biodistribution and whole-body scintigraphic studies. PSL supported and contributed intellectually to the project and carefully edited the manuscript. All authors read and approved the final manuscript.
